# A biogenic geodesic dome of the silica skeleton in Phaeodaria

**DOI:** 10.1038/s41598-024-64227-w

**Published:** 2024-06-12

**Authors:** Momoka Yamaguchi, Yasuhide Nakamura, Hiroto Watanabe, Katsunori Kimoto, Yuya Oaki, Shinji Shimode, Hiroaki Imai

**Affiliations:** 1 Department of Applied Chemistry, Faculty of Science and Technology, Keio University, 3-14-1 Hiyoshi, Kohoku-ku, Yokohama, 223-8522 Japan; 2 Estuary Research Center, Shimane University, 1060 Nishikawatsu-cho, Matsue-shi, Shimane, 690-8504 Japan; 3 Japan Agency for Marine-Earth Science and Technology (JAMSTEC), Natsushima-cho 2-15, Yokosuka, 237-0061 Japan; 4 Manazuru Marine Center for Environmental Research and Education, Graduate School of Environment and Information Sciences, Yokohama National University, 61 Iwa, Manazuru, 259-0202 Japan

**Keywords:** Structural biology, Biomaterials

## Abstract

Unique architectures of microbial skeletons are viewed as a model for the architectural design of artificial structural materials. In particular, the specific geometric arrangement of a spherical skeleton 0.5–1.5 mm in diameter of shell-bearing protists, Phaeodaria (*Aulosphaera* sp.), is remarkably interesting because of its similarity to a geodesic polyhedron, which is a hollow framework with 6-branched nodes that requires minimal building material for maximal strength. A phaeodarian skeleton composed of silica rods 5–10 µm in diameter was characterized as a distorted dome that is based on an icosahedron sectioned with a 7-frequency subdivision. The major difference of the biogenic architecture from the ideal geodesic dome is the coexistence of 7- and 5-branched nodes with the distortion of the frames and the presence of radial spines. From a microscopic perspective, the frames and radial spines were revealed to be hollow tubes having inner fibers and lamellar walls consisting of silica nanoparticles 4–8 nm in diameter with interlayer organic matter. The high degradability of the silica skeleton in seawater after cell mortality is ascribed to the specific nanometric composite structure. The biological architectonics sheds light on the production of environmentally friendly, lightweight structural materials and microdevices.

## Introduction

In nature, organisms produce a variety of inorganic substances having precisely controlled morphologies from a limited selection of ubiquitous elements in environmental conditions. The architectonics of biogenic skeletons is a critically important aspect of biological mineralization with regard to the emergence of specific vital functions. Moreover, several interesting biogenic structures inspire widespread applications in artificial systems^1,2^. Biominerals have been revealed to have hierarchical architectures that are built up of nanoscale grains incorporated with organic polymers, regardless of crystalline and non-crystalline states^3–7^. Specific hierarchical textures provide excellent mechanical properties of biominerals with characteristic morphologies^8–16^. Macro- and microscopic studies on the morphological design of skeletons are necessary for understanding the unique properties of biological materials. For instance, a hierarchical biological microlattice in the calcareous skeleton of a starfish, which exhibits lattice-level structural gradients and dislocations and the atomic-level conchoidal fracture behavior, enhances the damage tolerance^15^. Here, we focus on the geometric arrangement of a siliceous skeleton of a species of Phaeodaria (*Aulosphaera* sp.), shell-bearing protists, that is similar to a geodesic polyhedron because clarification of the total architectonics–including macro- and microscale structures–would contribute to the development of emergent artificial materials that have a framework requiring minimal building material for maximal strength.

Biogenic amorphous silica (SiO_2_･*n*H_2_O) is a typical biomineral that is widely observed in a variety of living organisms, including diatoms^5,17–22^, sponges^23–26^, skeletal protists^27,28^, and some higher plants^29–32^. The major organisms that produce biogenic silica in the ocean are categorized as sponges, diatoms, radiolarians (polycystines), and phaeodarians. Since the structures, morphogenetic mechanism, and functions of diatoms and sponges have been studied by many researchers^5,18,20,33–44^, silica frustules and spicules have been found to have adequately designed hierarchical architectures^25,17,18^ that provide excellent mechanical and optical properties^24,29,32,45–47^. An individual silica frustule produced in a silica deposition vehicle is a cell wall consisting of two valves held together by girdle bands^48,49^. Spicules in sponges are formed around an axial filament consisting of specialized silica deposition proteins^50^. The silicic skeleton of deep-sea hexactinellid sponges, such as *Euplectella aspergillum*, consists of a square-grid-like architecture overlaid with a double set of diagonal bracings^16^. The highest buckling resistance is achieved by the diagonal reinforcement strategy as the optimum material design for a given amount of material.

Research works on the siliceous skeletons of shell-bearing protists, such as phaeodarians and radiolarians, have been limited despite their great importance from the perspectives of marine biology and geology^27,28,51–53^. In particular, phaeodarian skeletons have not been sufficiently characterized because the porous frameworks are easily discarded in seawater after cell mortality. Under the current classification scheme, the subclass Phaeodaria is divided into 7 orders and 18 families. *Aulosphaera* sp. is a major component of the plankton community in several sea areas around the Japanese Archipelago^54,55^. The geometrically shaped skeleton is composed of triangular units consisting of hollow silica rods. However, further characterization is required to clarify detailed structures of the specific skeletons.

In the present study, we characterized the macroscale framework and microscale structures of the siliceous skeleton of *Aulosphaera* sp. to provide essential information for the architectonics of biological minerals that have specific geometric features. We analyzed the macro- and microscopic structures of *Aulosphaera* sp. to illuminate the geodesic architectonics of the specific framework of the skeleton with their mechanical functions and chemical properties. The phaeodarian spherical skeletons are found to be similar to geodesic structures that are a framework requiring minimal building material for maximal strength^56^. The popular frameworks consisting of equilateral triangles are based on an icosahedron in which each face is divided into triangles with a frequency (*f*) that describes the number of subdivisions of each face of the base polyhedron (Fig. S1 in the supporting information (SI)). However, the similarity of the geodesic structure to phaeodarian spheres has not been studied with quantitative evaluation. Here, the macroscale geometric frameworks were investigated using microfocus X-ray computed tomography (CT), scanning electron micrography (SEM), and mechanical simulation. Moreover, the microscale morphologies including internal structures were characterized by electron micrography, elemental analysis, and Raman spectra to shed light on the high degradability of the siliceous skeleton. Finally, we succeeded in accurately describing the architectonics, including the specular geometric arrangement and mesoscopic structural nature of the biological silica.

## Results and discussion

### Similarities and differences between the biogenic framework and ideal geodesic polyhedrons

**Figure 1:**
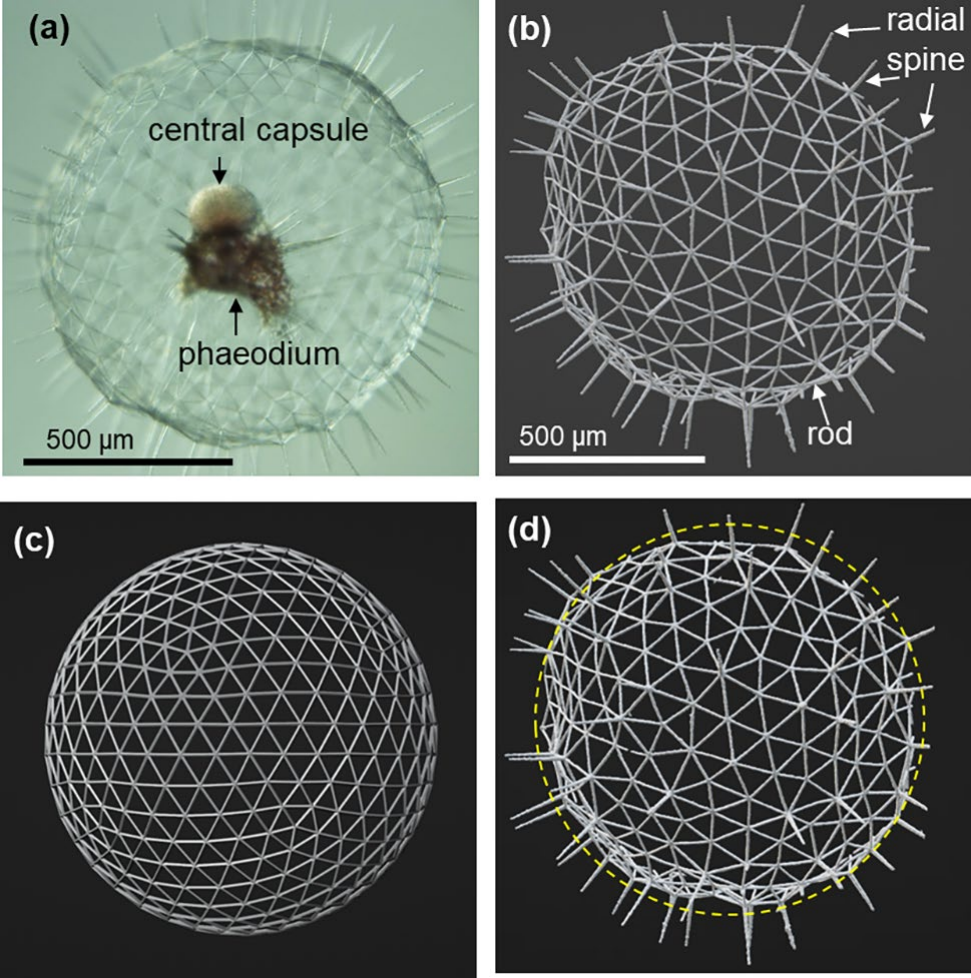
Geometrically arranged skeletons of the genus *Aulosphaera*. (**a**) Optical microscope and (**b**) microfocus X-ray CT images. (**c**) A 3D image of a geodesic dome that is based on the transformation of an icosahedron into a sphere in which each plane face is sectioned with a 7-frequency subdivision (*f*-7 geodesic dome). (**d**) A schematic image of the differences between the outlines of a biogenic dome and the ideal sphere of a geodesic dome.

**Figure 2:**
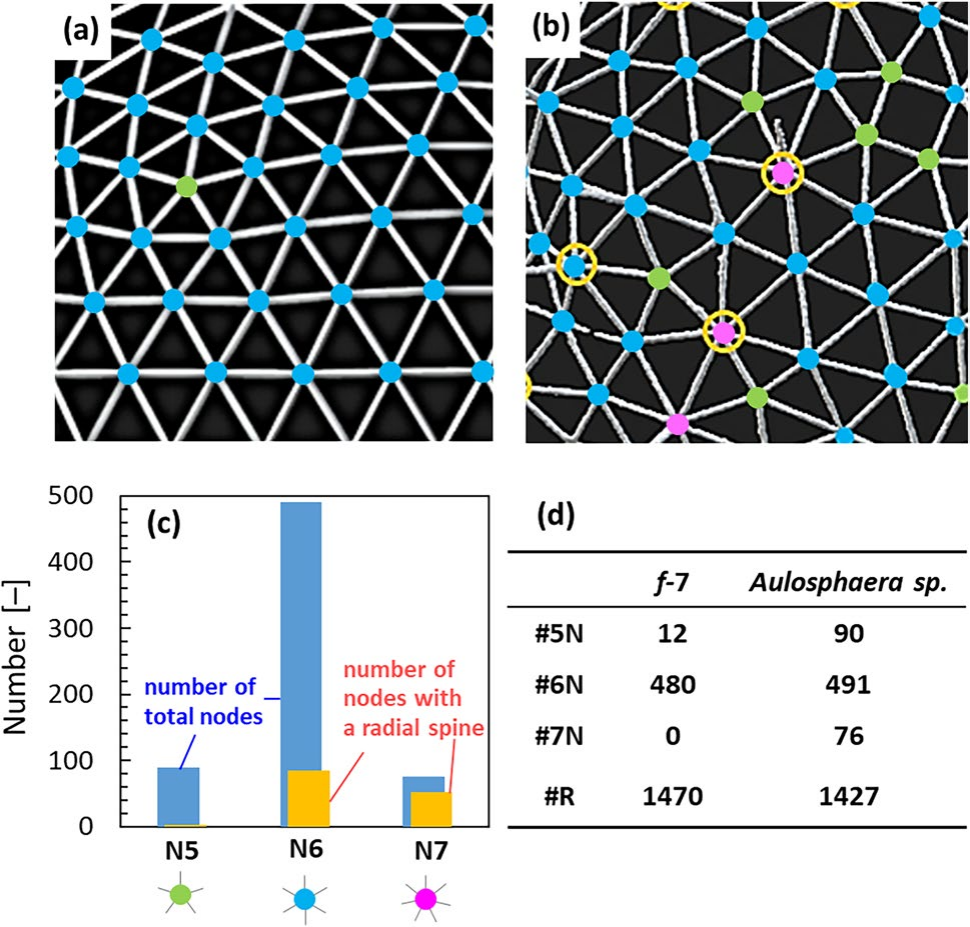
Enlarged images of frameworks of (**a**) a geodesic dome with a 7-frequency subdivision (*f*-7 geodesic dome) and (**b**) the biogenic skeleton. (**c**,**d**) A histogram and a list of numbers of three kinds of nodes having 5- (N5), 6- (N6), and 7- (N7) branches in the *f*-7 geodesic dome and the biogenic skeleton. Green, blue, and pink dots indicate N5, N6, and N7, respectively.

Figure 1 shows an optical micrograph of a living body and a microfocus X-ray CT image of a whole skeleton of *Aulosphaera* sp. The body has a central capsule, a phaeodium, and a geometrically shaped skeleton that is covered with a transparent organic veil (Fig. 1a). The skeleton is basically composed of a triangle framework consisting of thin rods with radial spines (Fig. 1b). We show a 3D image of an icosahedron in which each plane face is sectioned into a network of smaller triangles with a 7-frequency subdivision (*f*-7 geodesic dome) as a reference (Fig. 1c). The presence of radial spines as pillars supporting the expansion of an organic veil is a feature characteristic of the biogenic dome (Fig. S2 in the SI). The detailed arrangement of the skeletal framework is displayed in the microfocus X-ray CT images for three samples (Fig. S3 in the SI) for comparison with a geodesic polyhedron based on the layout of an icosahedron transformed into a sphere. As shown in Fig. 2 and Fig. S3 in the SI, the biogenic skeleton is composed of three kinds of nodes having 5- (N5), 6- (N6), and 7- (N7) branches, whereas the geodesic dome is mainly comprised of N6 with a fixed number (12) of N5. The numbers of rods (#R) and N6 in a geodesic polyhedron increase with increasing *f* (Fig. S1 in the SI).

As shown in Figs. 1 and 2c,d and Figs. S1 and S3 in the SI, both the biogenic frame and the *f*-7 geodesic dome are spherical structures composed of almost the same numbers of rods. On the other hand, the numbers of rods in the *f*-6 and *f*-8 domes are smaller and larger than that in the biogenic frame, respectively. We defined the frequency of subdivision from the similarity of the rod number in the geodesic domes.

The average rod length was almost the same regardless of the skeleton size (Fig. S3 in the SI). Here, we found that a major difference between the biogenic skeleton and the geodesic dome is the presence of excess numbers of N5 and N7 with slight variations in the vertex angles and rod lengths. The most interesting feature of the biological structure is the presence of N7. The number of N5 of the biological skeleton is much greater than 12 and is almost the same as that of N7. As shown in Fig. 2c and Fig. S3 in the SI, the basal nodes supporting the radial spines are N6 and N7. Almost every N7 has the radial spine, although radial spines were hardly observed on N5.

Pair generation of N5 and N7 is known to be induced with deformation of geodesic structures^57,58^. As shown in Fig. S3(c) in the SI, the formation of N5 is needed to compensate for the strain of the presence of N7 in the framework consisting of N6. Actually, most N5 are located near N7 (Fig. 2b and Figs. S3 and S4 in the SI). Since the biogenic skeleton is deformed (Fig. 1d), almost the same numbers of N5 and N7 are assumed to be formed as a pair in the framework. By assuming that two pairs of N5 and N7 replaced by four N6, revised numbers were calculated as shown in Fig. S3 in the SI. Since the revised numbers of N5 and N6 (#N5’ and #N6’, respectively) and #R are close to #N5, #N6, and #R of the 7-*f* geodesic dome, respectively, the biogenic skeleton is based on the layout of an icosahedron into a sphere by sectioning with a 7-frequency subdivision.

**Figure 3:**
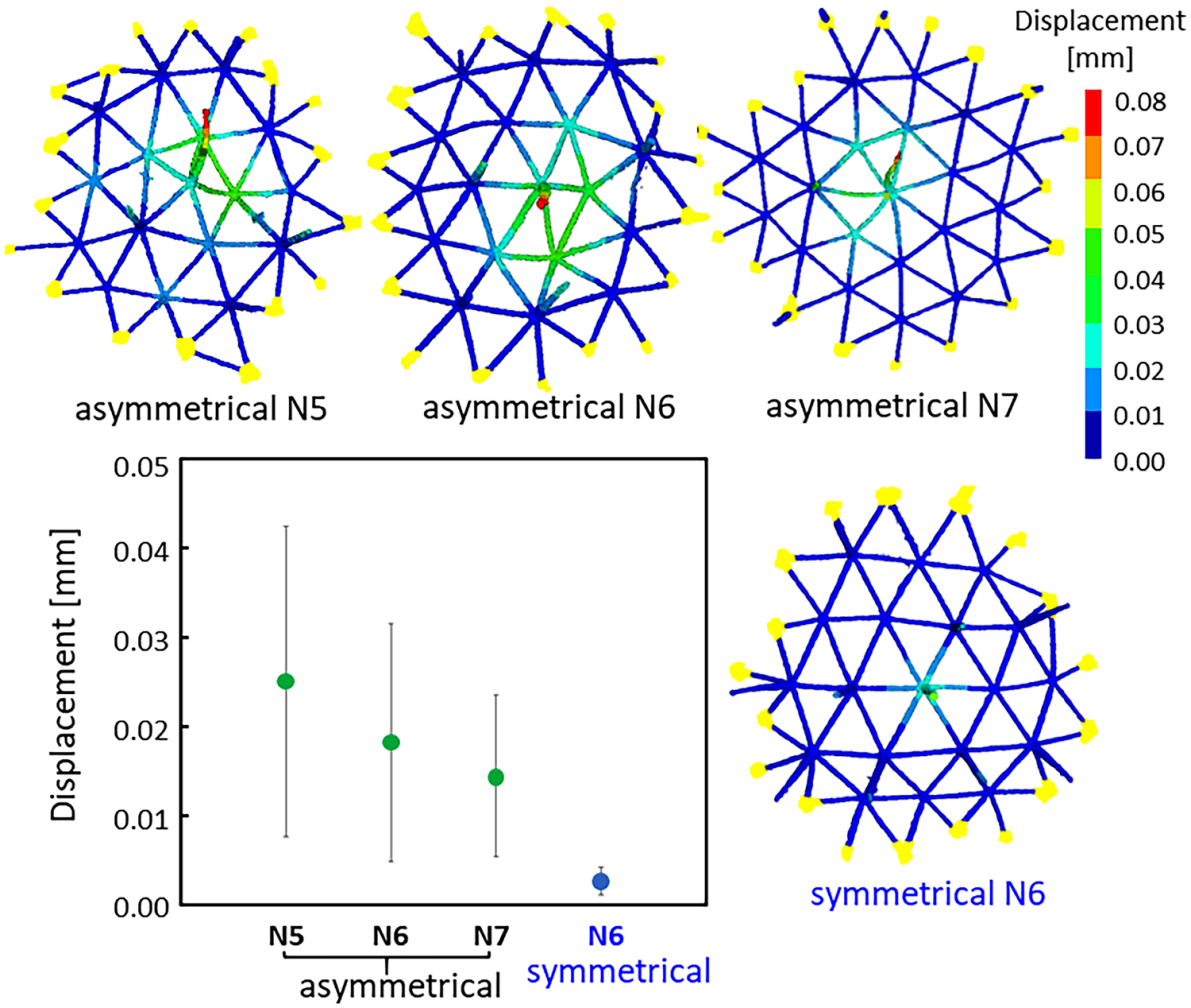
Schematic images of the calculated displacements of the frames around N5, N6, and N7. The average values and variation of the displacements of the node points around the radial spine.

The radial spines are utilized as pillars for the expansion of an organic veil covering the skeleton (Fig. S2 in the SI). Thus, the presence of many branches would be advantageous for the nodes to support the upper radial spine. The mechanical properties of the biological framework around the nodes were studied by numerical simulation using Voxelcon (Quint). From the CT images of the whole skeleton, we extracted small frameworks around N5, N6, and N7 that had radial spines. A numerical simulation was used to calculate the deformation of the frames around a radial spine with the application of a load on top of the radial spine. As shown in Fig. 3, displacements around the nodes can be compared by the change in color. The strain of symmetric N6 is smaller than that of asymmetric nodes including N5, N6, and N7. However, most of the frame structures around nodes are deformed and asymmetric in the biogenic skeleton (Fig. 2 and Fig. S3 in the SI). The smallest strain of the asymmetric frameworks is N7, followed in order by N6 and N5. These results suggest that asymmetric N7 has a relatively high mechanical strength as a base of the radial spine in a disordered framework of a distorted dome. Thus, most of the radial spines are deduced to be located on N7 and N6 (Fig. 2c).

Geodesic structures are known as a framework requiring minimal building material for maximal strength. We demonstrated the mechanical property of the biogenic frame using a compression test. SEM images of Fig. S5 in the SI show a part of the frame before and after compression by an indenter. Although the frame was deformed by the tip of the indenter, the basic triangle structure was not crushed. This indicates the robustness of the geodesic dome in the biogenic silicic skeleton.

### Microscopic morphology and internal structure of rods and radial spines

**Figure 4:**
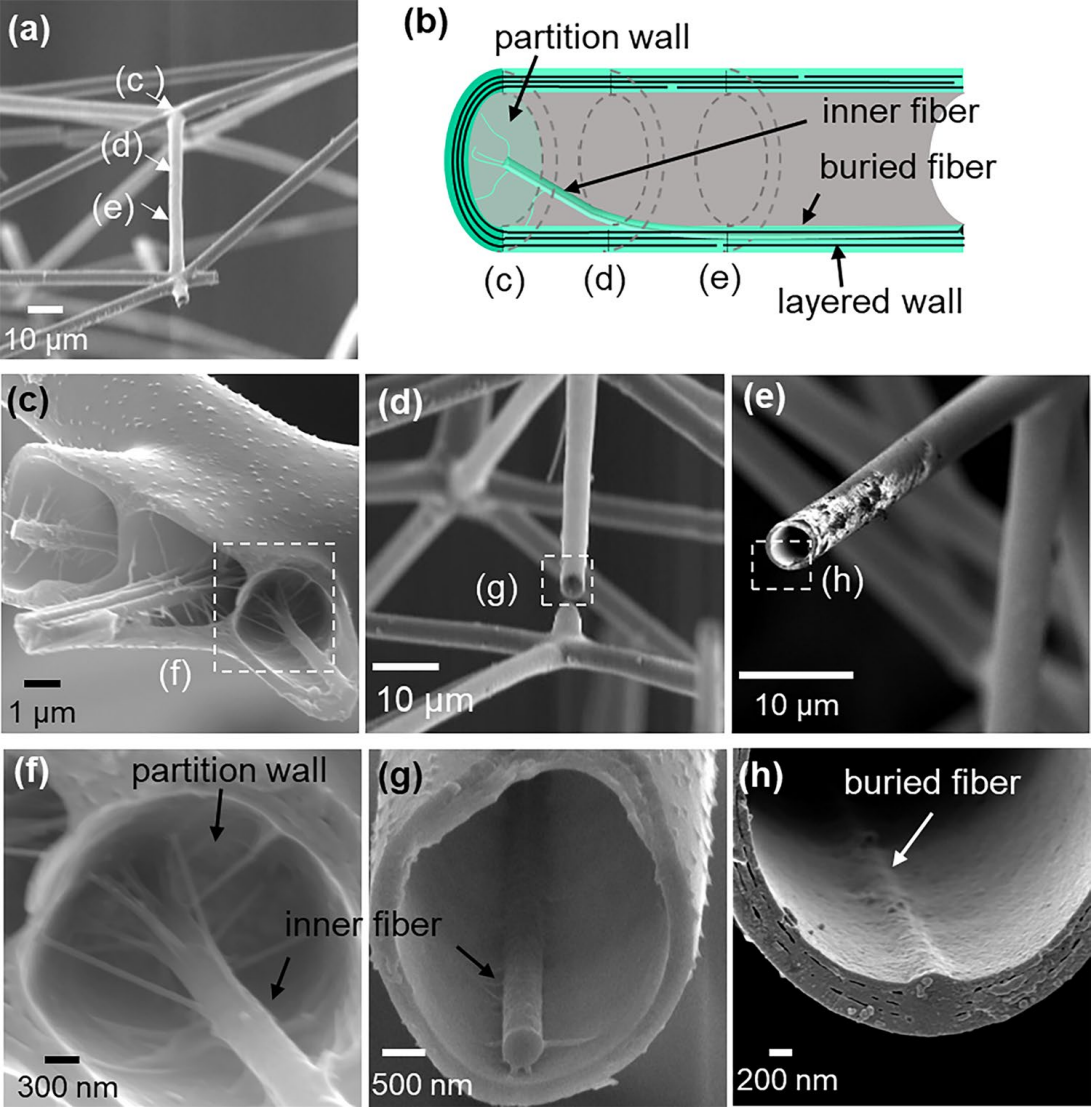
SEM images (**a**,**c**–**h**) and schematic illustration (**b**) of the rods. (**a**) A whole image, (**c**–**e**) cross-sectional images of a rod in (**a**), and (**f**–**g**) enlarged images in (**c**–**e**).

The rods of the phaeodarian skeleton were reported to be hollow tubes consisting of layered silica walls^28^. However, details of the internal structures of the rods have not been clarified. In the present work, the microscopic inside morphology of the rods, radial spines, and nodes was adequately characterized by SEM with and without chemical and heat treatments. Energy-dispersive X-ray spectroscopy (EDS) revealed that the rods consist of silica (Fig. S6 in the SI). Figure 4 shows cross-sectional SEM images and schematic illustration of the rods consisting of a lamellar wall 200–300 nm thick. The tubular walls are made of stacking of 3–4 layers. Here, we found partition walls and inner fibers 200–300 nm in diameter as internal structures (Fig. 4f,g). The rods are separated from the nodes by partition walls. The inner fiber near the nodes is connected to the center of the partition wall by thin fibrils. On the other hand, the fibers are eccentrically cramped and partially buried in the wall in the middle region of the rod (Fig. 4h). Most fibers are fixed in the side near the central capsule (Fig. S7 in the SI).

**Figure 5:**
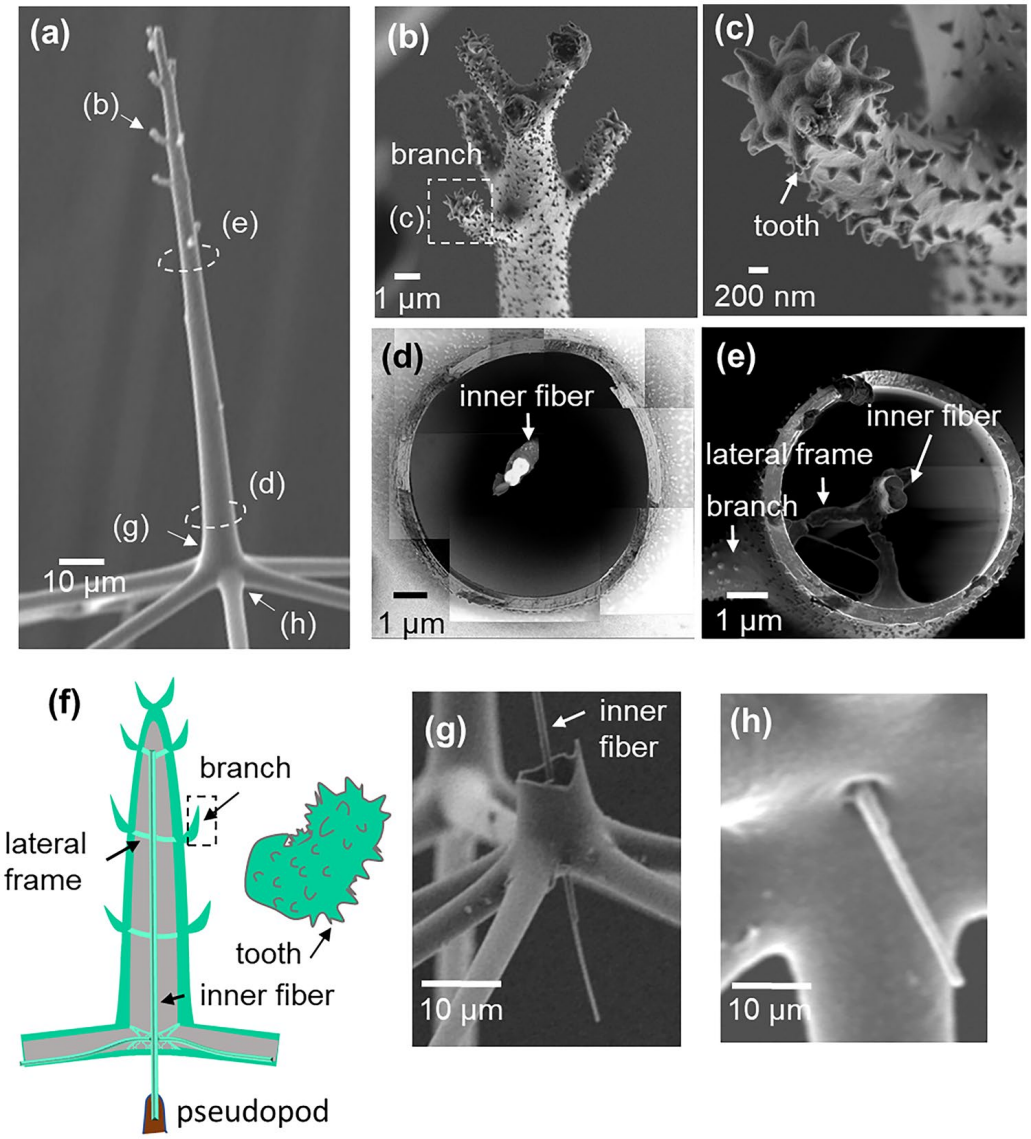
SEM images (**a**–**e**,**g**,**h**) and schematic illustration (**f**) of the appearance and the cross-sectional structure of the radial spines. (**a**) A whole image of a radial spine, (**b**,**c**) enlarged images of a branch (**d**) a cross section of a radial spine, (**e**) a cross section of a radial spine with branches, and (**g**,**h**) enlarged images of the node under a radial spine.

Figure 5 and Fig. S8 in the SI show SEM images and a schematic illustration of the appearance and the cross-sectional structure of the radial spines. The radial spines are tapered tubes (Fig. 5a,f) with a wall 200–400 nm thick (Fig. 5d,e). The diameter and the wall thickness of the basal part of radial spines are greater than those of the rods. The tubular walls of the radial spines have a lamellar structure of silica which is similar to that of the rod walls. The number of layers from ~5 to ~2 of the wall thickness decreases from the base to the top. An inner fiber was found to be fixed in the center of the radial spine (Fig. 5d–f) and penetrated the node as a radial spine base (Fig. 5g,h). The main component of the fibers was assigned to silica by the elemental mapping for the underside of a node (Fig. S9 in the SI). Branches having small teeth are attached on the upper part of the outside (Fig. 5a–c). The central fiber is connected to the branches with lateral frames (Fig. 5e,f).

**Figure 6:**
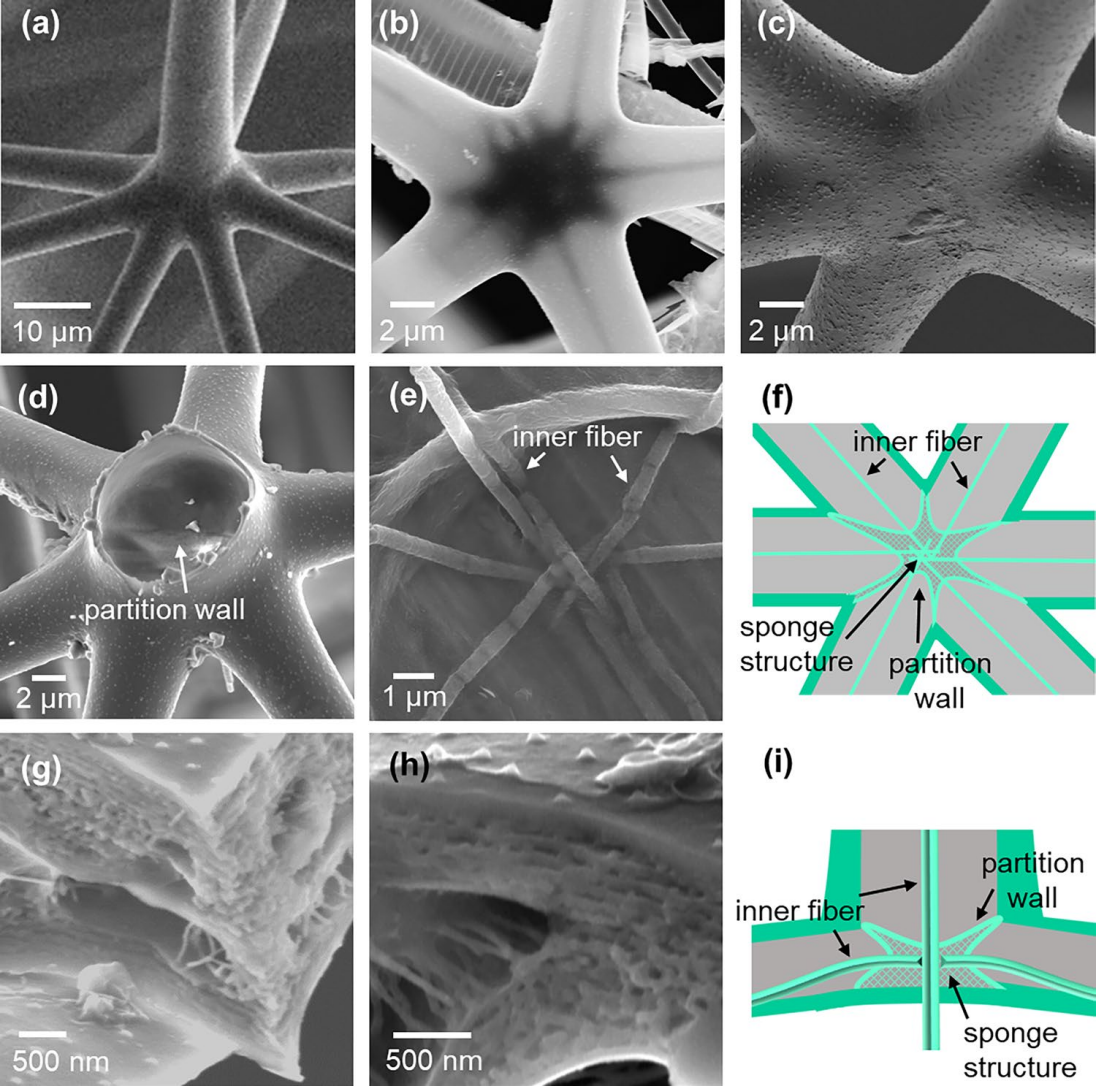
SEM (**a**,**c**–**e**,**g**,**h**), backscattered electron images (**b**), and schematic illustration (**f**,**i**) of the appearance (**a**–**c**) and lateral cross sections (**d**–**f**) and vertical cross sections (**g**–**i**) of the node.

Figure 6 shows SEM images and schematic illustrations of the appearance and the cross-sectional structure of the nodes. As described above, the rods and radial spines have hollow tubular structures. Thus, the biogenic framework is an efficient structure for expending minimal building material for maximal strength even from a microscopic prospective. On the other hand, the nodes are suggested to have an internal structure from the shadow at the node in a backscattered electron image (Fig. 6b). The space of the node is separated from the rods and the radial spine by walls. A sponge structure was observed in the center of the nodes (Fig. 6g–i). The connecting points of the framework would be strengthened by the internal structures of the nodes. The inner fibers in the rods are connected to each other in the node. Under the node attached with the radial spine, the inner fiber is gripped by pseudopods under the radial spines (Fig. 5f and Fig. S10 in the SI). Thus, the fibers can be utilized as a signal transmission system.

### Nanometric analysis of the silica walls of rods and radial spines

**Figure 7:**
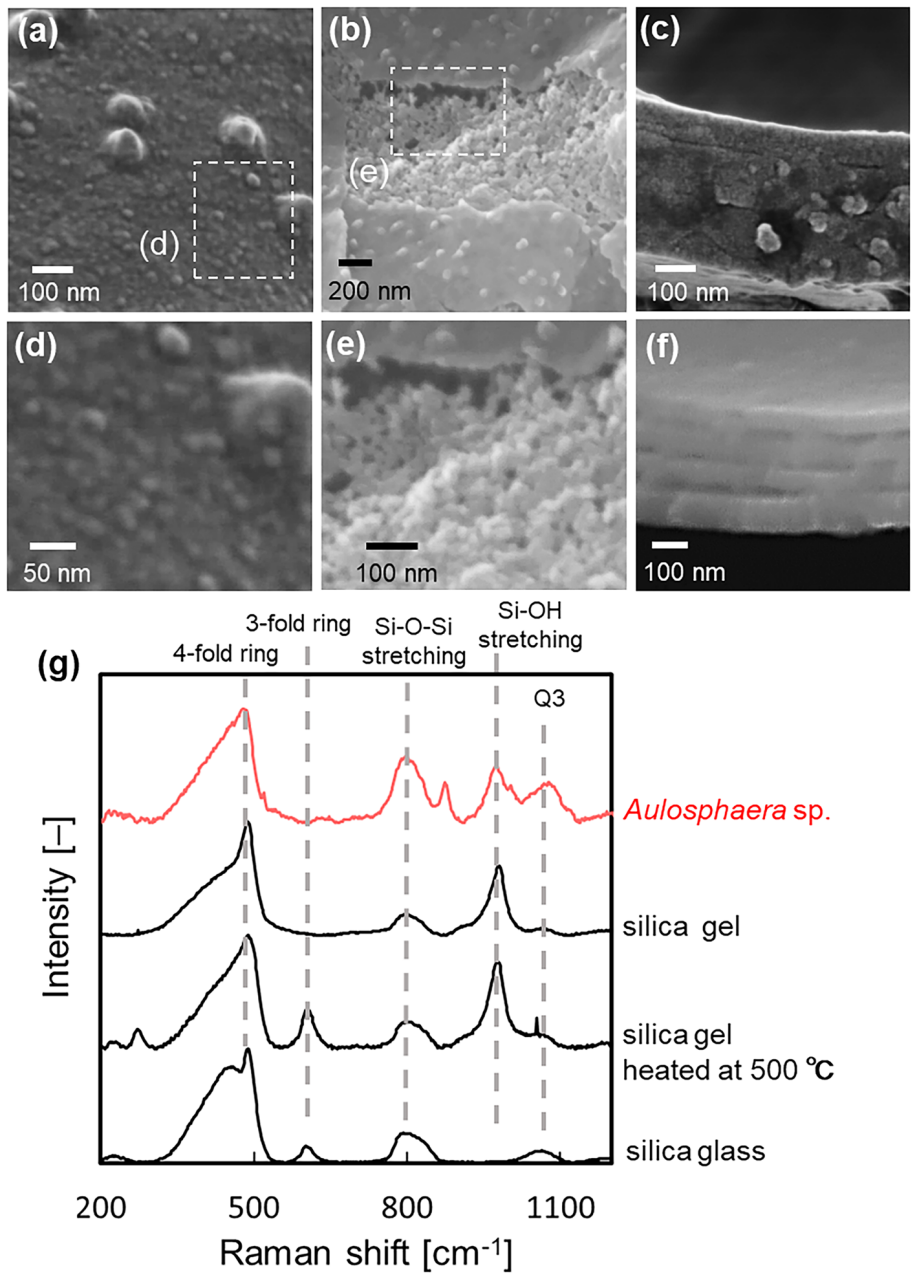
SEM images (**a**–**f**) of the rod wall and Raman spectra (**g**) of the skeleton and various kinds of silicas. The rod surfaces before (**a**,**d**) and after (**b**,**e**) storage in seawater and subsequent firing at 500 °C in air. The rod cross section before (**f**) and after NaClO treatment for 48 h.

Figure 7 shows SEM images of the surface and a cross section of the rod wall. Nanoparticles were observed on the outer and cross-sectional surfaces of the rod walls (Fig. 7a,c,d). The nanograins were clearly observed after the skeleton was stored in seawater for several days and after firing at 500 °C in air (Fig. 7b,e). These results indicate that the skeleton is composed of nanoparticle 4–8 nm in diameter. As reported in a previous work, the wall is composed of several layers^28^. After NaClO treatment for 48 h, we observed spaces between the layers (Fig. 7f). EDS mapping images showed that the amounts of carbon in the inside layers are higher than that in the outer coat (Fig. S11 in the SI). Organic matter is deduced to exist in the interlayer spaces. Since the residue after the dissolution of silica was colored with Coomassie brilliant blue, the organic matter contains large amounts of amino acids (Fig. S12 in the SI).

Raman spectra for the skeleton are compared to those of a commercial silica gel before and after calcination at 500 °C (Fig. 7g). Basically, the skeleton is deduced to be composed of amorphous silica from the spectra. However, sharp signals assigned to planer 3- and 4-fold rings are not observed for the skeleton^59^. Moreover, an intense signal due to tertiary (Q3) SiO_4_ tetrahedra, which are assignable to a silicate structure having a dangling bond, is a feature of the Raman spectra. Therefore, the linear chains of the Si–O bands are dominant with a large amount of OH groups, whereas regular silica glass is comprised of ring structures. The skeleton consisting of nanoparticles with a large amount of organic matter was degraded in natural seawater after cell mortality. Moreover, the silica network is composed of linear chains. Since the basicity increases with the decomposition of amino acids, the biogenic siliceous skeleton easily decomposes in a high pH aqueous solution.

### Comparison of the skeletal architecture of *Phaeodaria* with those of benthic organisms

We can find adequate design on skeletal geometry. Basically, the weight of the skeleton is not important for the biological activity of benthic organisms. Thus, a three-dimensional ordered lattice consisting of thick frames is adopted to improve the mechanical strength. For example, a hierarchical biological microlattice in the calcareous solid skeleton of the knobby starfish enhances the damage tolerance^15^. The skeleton of deep-sea hexactinellid sponges, such as *Euplectella aspergillum*, is basically composed of thin silicic fibers to save the materials to trim weight and save the material^16^. However, the buckling resistance of a simple square-grid-like architecture with nodes having 4 branches is not sufficient for mechanical irritation. Thus, the diagonal reinforcement strategy, such as a double set of diagonal bracings, is needed as the optimum material design for a given amount of material.

The skeleton of planktonic organisms requires weight saving to float in the water. The geodesic architectures consisting of triangles with thin rods and nodes having 6 branches are an ideal design to achieve maximal strength using minimal building material. Thus, the skeleton of a large plankton, such as Phaeodaria, is based on the geodesic frames with silicic hollow fibers. Moreover, the variation of the branching structure of the frames is modified to install radial spines. Finally, a light-weight strong skeleton is designed for the vital activity of the plankton.

## Conclusion

The specific macroscale arrangement and microscale structure of the skeleton of Phaeodaria (*Aulosphaera* sp.) were clarified using microfocus X-ray computed tomography, electron micrography, elemental analysis, Raman spectra, and mechanical simulation. The geometric body of the siliceous skeleton of Phaeodaria composed of silica hollow rods ~ 1 µm in diameter is geometrically similar to a geodesic polyhedron based on the layout of an icosahedron sectioned with a 7-frequency subdivision. The major difference of the biogenic architecture from the ideal geodesic dome is the coexistence of 7- and 5-branched nodes with a distortion of the frames and the presence of radial spines as pillars supporting the expansion of an organic veil. The frames that include radial spines were revealed to be hollow tubes that have inner fibers and layered walls consisting of silica nanoparticles 4–8 nm in diameter with interlayer organic matter. We can design environmentally friendly, lightweight microdevices from the biological architectonics.

## Methods

Plankton were sampled at 35° 09.45′N, 139° 10.00′E in the western part of Sagami Bay, Japan on R/V Tachibana of the Manazuru Marine Center for Environmental Research and Education, Yokohama National University. Plankton were collected by plankton nets (diameter: 80 cm, side length: 3 m, mesh size: 100 μm or diameter: 45 cm, side length: 1.8 m, mesh size: 180 μm). From the plankton samples, individuals of *Aulosphaera* sp. were picked up and stored in ethanol (in seawater, which was replaced once a day when checking degradability) and observed after washing in pure water.

Sodium hypochlorite at pH 5 was used to remove organic matter. The overall view of *Aulosphaera* sp. was observed by microfocus X-ray CT, SEM and optical microscopy. Shell morphometry of *Aulosphaera* sp. was performed by microfocus X-ray CT (ScanXmateD160TSS105, Comscantechno Co.) equipped by JAMSTEC. A high-resolution setting (X-ray focus diameter: 0.8 µm; X-ray tube voltage: 70 keV; X-ray tube current: 85 µA; detector array size of 1024 × 1024 pixels; 2000 projections in 360° rotations) was used. The geometric resolution of the isotropic voxel size was from 0.28 to 0.46 µm/voxel. We used coneCTexpress (White Rabbit Corp.) software for correction and reconstruction tomography data and the general principle of Feldkamp’s cone-beam reconstruction was followed to reconstruct image cross sections based on filtered back projections. The surfaces and cross sections of the samples were coated with osmium for detailed observation using a scanning electron microscope (SEM, FEI Helios G4 UX, JEOL JSM-7100, Zeiss Merlin Compact) operated at 1.0–10.0 kV. The compositions were identified using Raman scattering spectroscopy and energy-dispersive X-ray analysis (JEOL JED-2300). Micro-Raman spectroscopy was performed using a laser confocal microscope (inVia, Renishaw). The 532 nm excitation laser was focused on the sample surface with a 100× objective lens of the microscope. The size of the laser spot was approximately 1 μm in diameter. Silica gel and silica glass used as references were purchased from Kanto Chemical and Hamamatsu Photonics, respectively. Coomassie Brilliant Blue (FUJIFILM Wako, 038-17932) and HF treatments were performed according to a previous work^24^.

After a series of these treatments, the examined specimens were taxonomically identified under the modern classification concept and named following the International Code of Zoological Nomenclature. Voxelcon (Quint) was used for the dynamics simulation; cubes (voxels) were added to the stl data surface and fleshed out. The size of one voxel was set to be 1.64×10^-3^ mm. Poisson's ratio, Young's modulus, and magnitude of load were set to 0.35, 0.7 GPa, and 1 mN, respectively. The physical property values were referred to those of typical thermoplastic polymers.

### Supplementary Information

Supplementary Information.

## Data Availability

The authors declare that the data supporting the findings of this study are available within the paper and its Supplementary Information file. Raw data files are available from the corresponding author upon reasonable request.
