# Supplementary material for: A biogenic geodesic dome of the silica skeleton in Phaeodaria
Source: Sci Rep. 2024 Jun 12;14:13481. doi: 10.1038/s41598-024-64227-w (PMC11169525; doi:10.1038/s41598-024-64227-w)
Supplement: Supplementary file 1 — Supplementary Information. [file 41598_2024_64227_MOESM1_ESM.pdf]

A biogenic geodesic dome of the silica skeleton in Phaeodaria

Momoka Yamaguchi[1] • Yasuhide Nakamura[2] • Hiroto Watanabe[1] • Katsunori Kimoto[3] • Yuya Oaki[1] • Shinji Shimode[4] • Hiroaki Imai[1*]

[1]Department of Applied Chemistry, Faculty of Science and Technology, Keio University, 3-14-1 Hiyoshi, Kohoku-ku, Yokohama 223-8522, Japan.
[2]Estuary Research Center, Shimane University, 1060 Nishikawatsu-cho, Matsue-shi, Shimane 690-8504, Japan.
[3]Japan Agency for Marine-Earth Science and Technology (JAMSTEC), Natsushima-cho 2-15, Yokosuka 237-0061, Japan.
[4]Manazuru Marine Center for Environmental Research and Education, Graduate School of Environment and Information Sciences, Yokohama National University, 61 Iwa, Manazuru 259-0202, Japan.
E-mail[*]: hiroaki@applc.keio.ac.jp

p. S2. Schematic illustrations with a list of numbers of nodes and rods for a geodesic dome
An optical microscope image of *Aulosphaera* sp. expanding its organic veil
p. S3. Microfocus X-ray CT images for three samples with a list of numbers of nodes and rods and average lengths of rods in a phaeodarian skeleton.
p. S4. Microfocus X-ray CT images for three samples
p. S5. SEM images for a part of the frame before and after compression by an indenter
SEM and EDS images of the surface of a rod
p. S6. SEM images and a schematic illustration of cross sections of rods
Schematic illustrations and SEM images of the layered structure of the radial spine wall
p. S7. SEM and EDS images of the cross section of the fiber on the underside of a node
Optical microscope images of nodes that have a radial spine
p. S8. SEM and EDS images of a cross section and a side view of a radial spine
Optical microscope images of the framework after Coomassie brilliant blue staining and after subsequent HF treatment

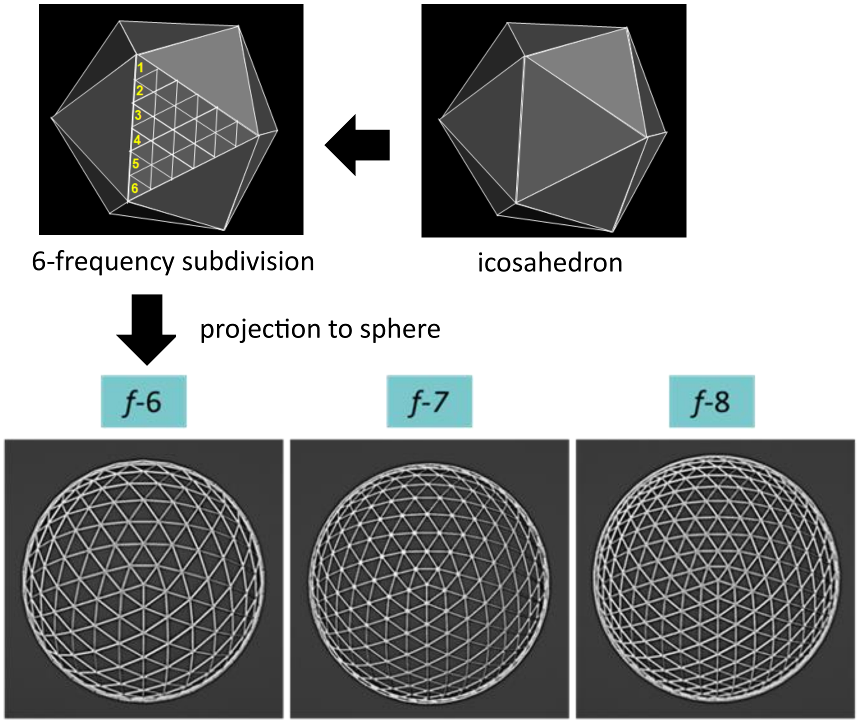

frequency (*f*)	6	7	8
#N5	12	12	12
#N6	350	480	630
#R	1080	1420	1920

Figure S1. Schematic illustrations with a list of numbers of 5- and 6-branched nodes (#N5 and #N6) and rods (#R) for icosahedrons and geodesic domes sectioned with *f* = 6, 7, and 8. The geodesic dome is produced by projection to a sphere of an icosahedron in which each face is divided into triangles with a frequency (*f*). The number of N5 (#N5) is fixed to 12 because N5 originates from the 12 peaks of the original icosahedron.

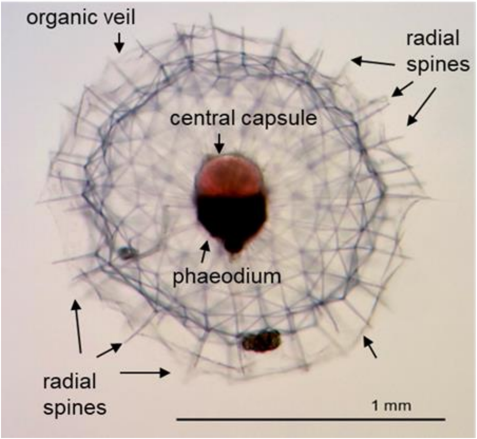

Figure S2. An optical microscope image of *Aulosphaera* sp. expanding its organic veil. Radial spines support the expanding veil as pillars.

(a)

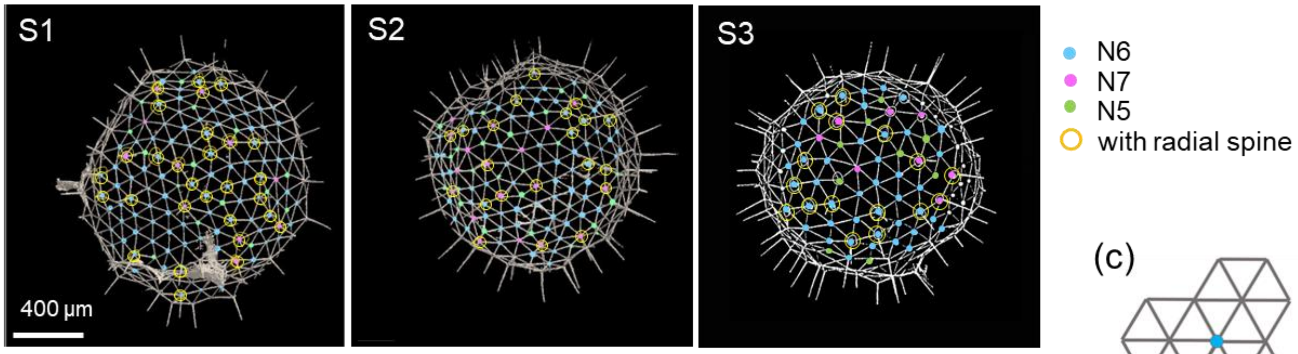

(b)

sample	S1		S2		S3	
	total	w/spine	total	w/spine	total	w/spine
**#N5 (#N5’)**	90 (14)	2	84 (12)	5	78 (22)	6
**#N6 (#N6’)**	491(643)	84	385(529)	75	313(425)	70
**#N7 (#N7’)**	76 (0)	52	72 (0)	50	56 (0)	47
**sum**	657	138	541	130	447	123
**#R**	1427		1360		1268	
**ave. of rod length [mm]**	0.095 ± 0.016 n = 312		0.099 ± 0.015 n = 248		0.100 ± 0.018 n = 253	
**diameter [mm]**	1.33 ± 0.07		1.21 ± 0.05		1.22 ± 0.05	

(c)

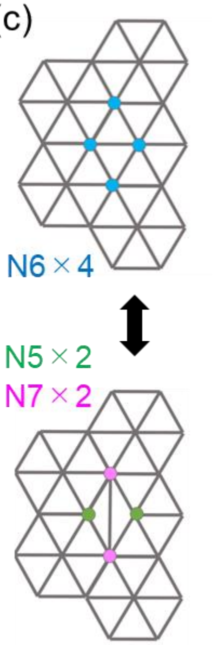

Figure S3. (a) Microfocus X-ray CT images (a) for three samples (S1, S2, and S3) with (b) a list of numbers of nodes and rods and average lengths of rods in a phaeodarian skeleton.
By assuming that two pairs of N5 and N7 are replaced by four N6 as shown in (c), revised numbers of N5 (#N5’) and N6 (#N6’) were calculated using the following equations.

#N5' = #N5–#N7
#N6' = #N6 + #N7×2

The revised number of N7 (#N7’) is fixed to zero. The similarity of #N5’, #N6’, and #R suggests that the biogenic skeleton is close to the *f*-7 geodesic dome.

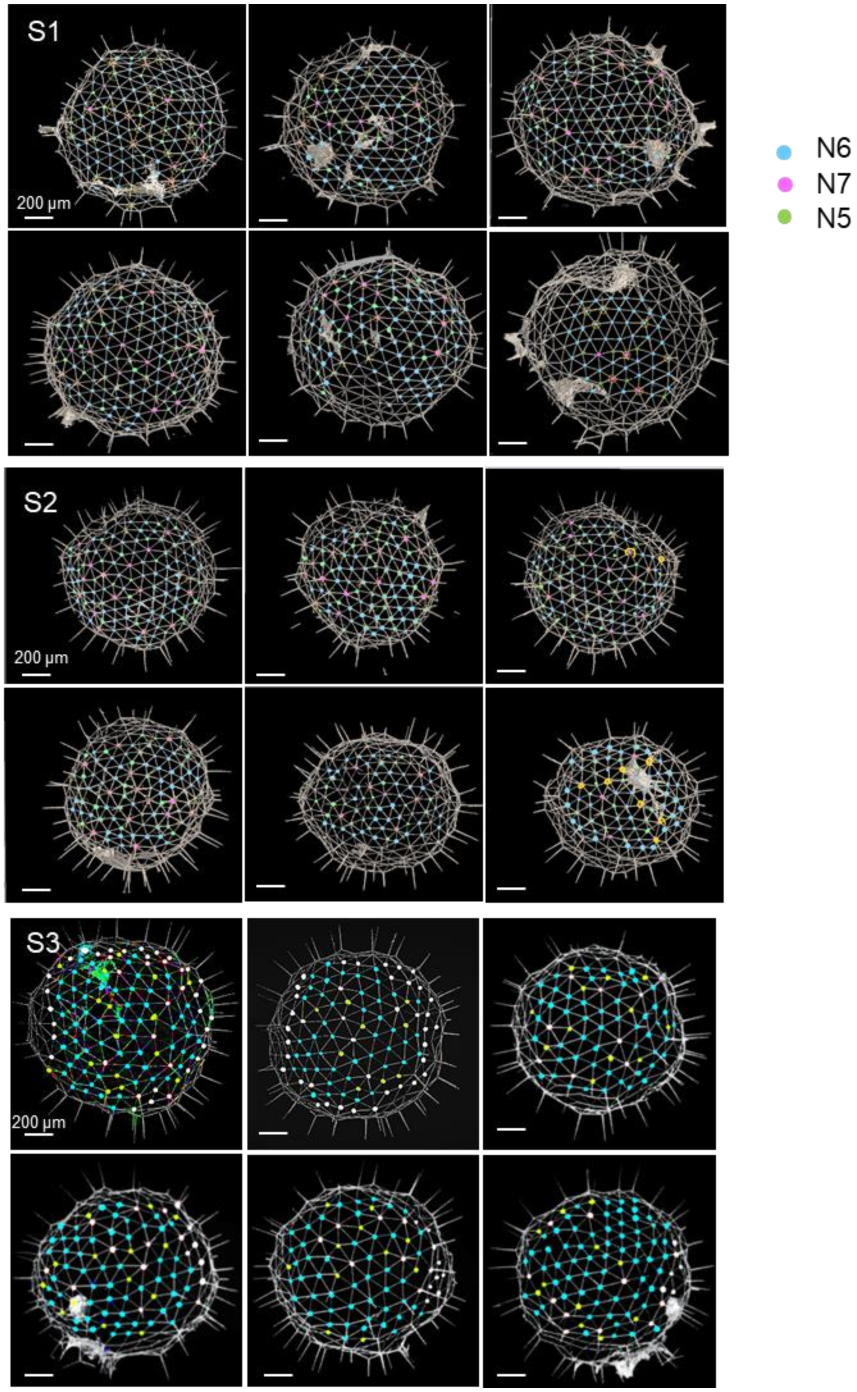

Figure S4. Microfocus X-ray CT images of three phaeodarian skeletons (S1, S2, and S3).

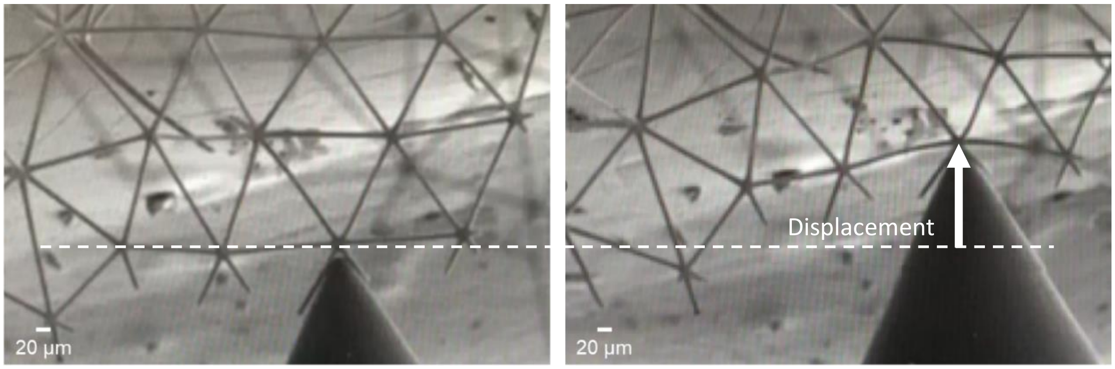

Figure S5. SEM images of a part of the frame before and after compression by an indenter. Although the frame was deformed by the tip of the indenter, the basic triangle structure was not crushed.

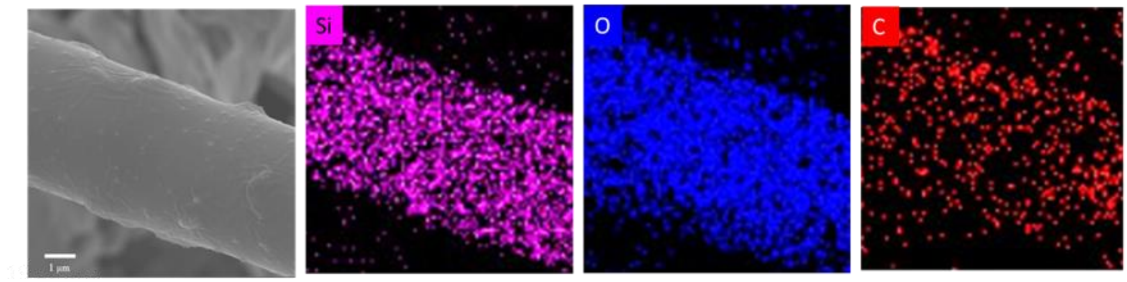

Figure S6. SEM and EDS images of the surface of a rod. The presence of silicon, oxygen, and carbon indicates that the rods consist of silica containing organic matte

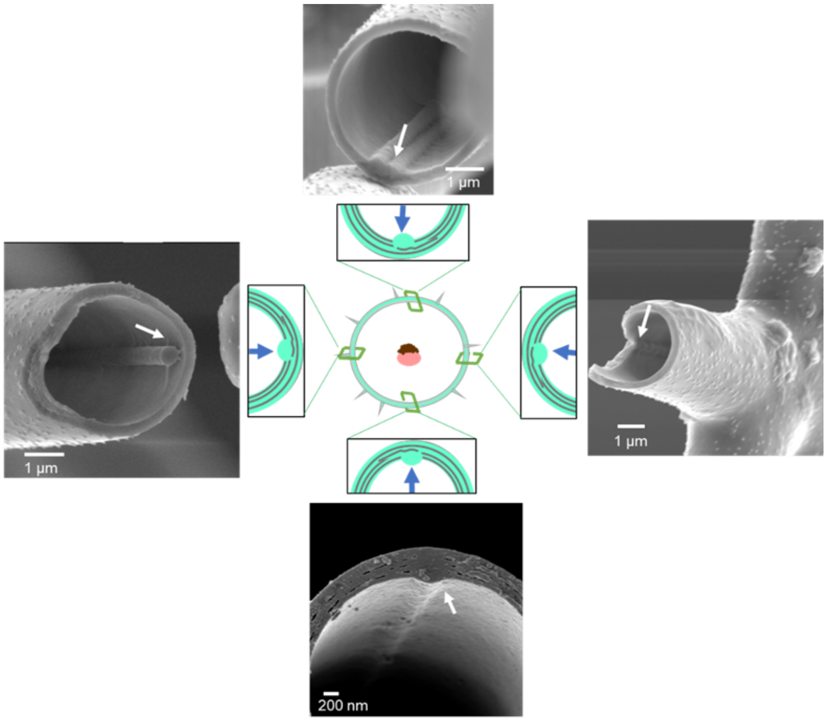

Figure S7. SEM images and a schematic illustration of cross sections of rods indicating the sides in which the inner fibers are cramped. Most fibers are fixed in the side near the central capsule.

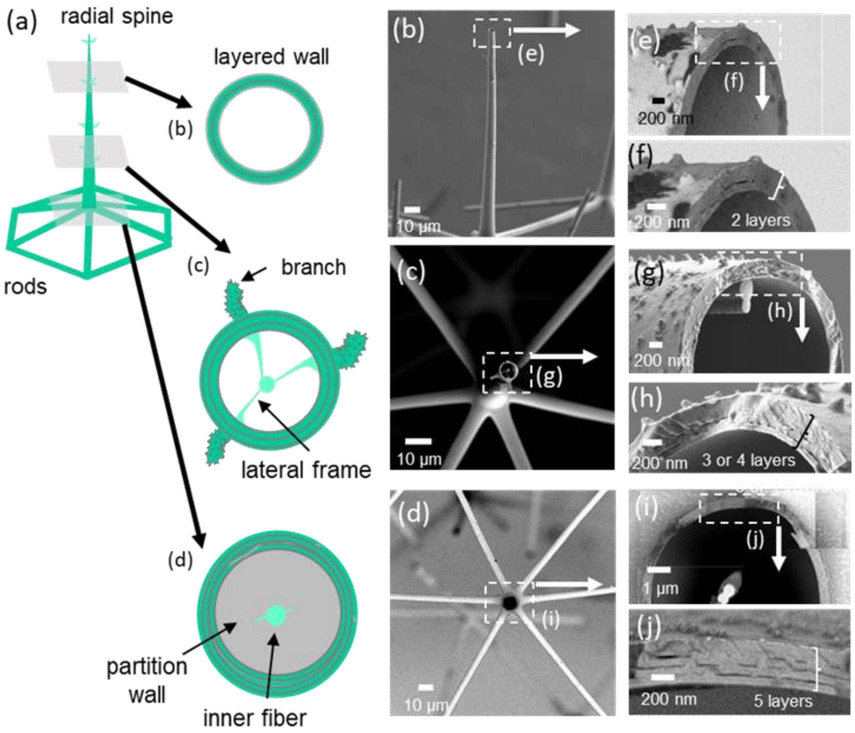

Figure S8. Schematic illustrations and SEM images of the layered structure of the radial spine wall.

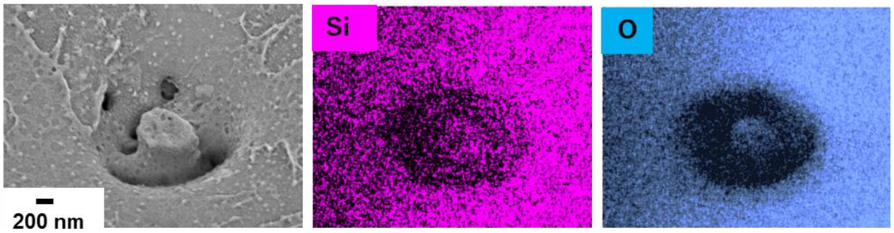

Figure S9. SEM and EDS images of a cross section of a fiber on the underside of a node. The presence of silicon and oxygen indicates that the fibers mainly consist of silica.

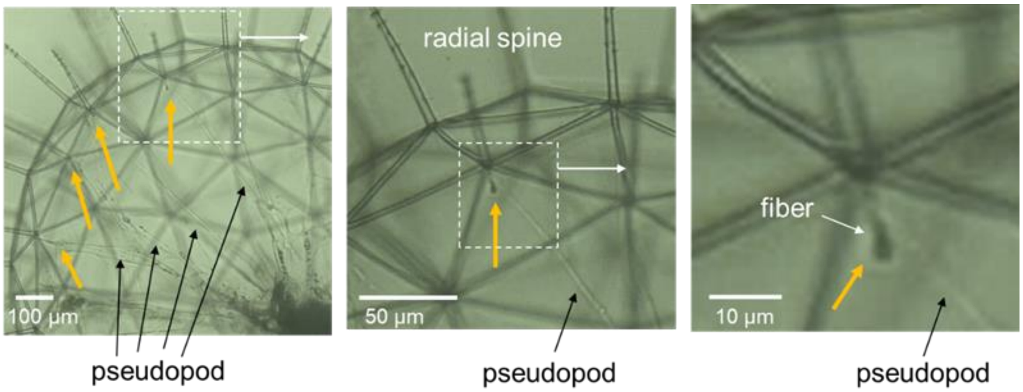

Figure S10. Optical microscope images of nodes that have a radial spine. Yellow arrows indicate the ends of fibers gripped by pseudopods.

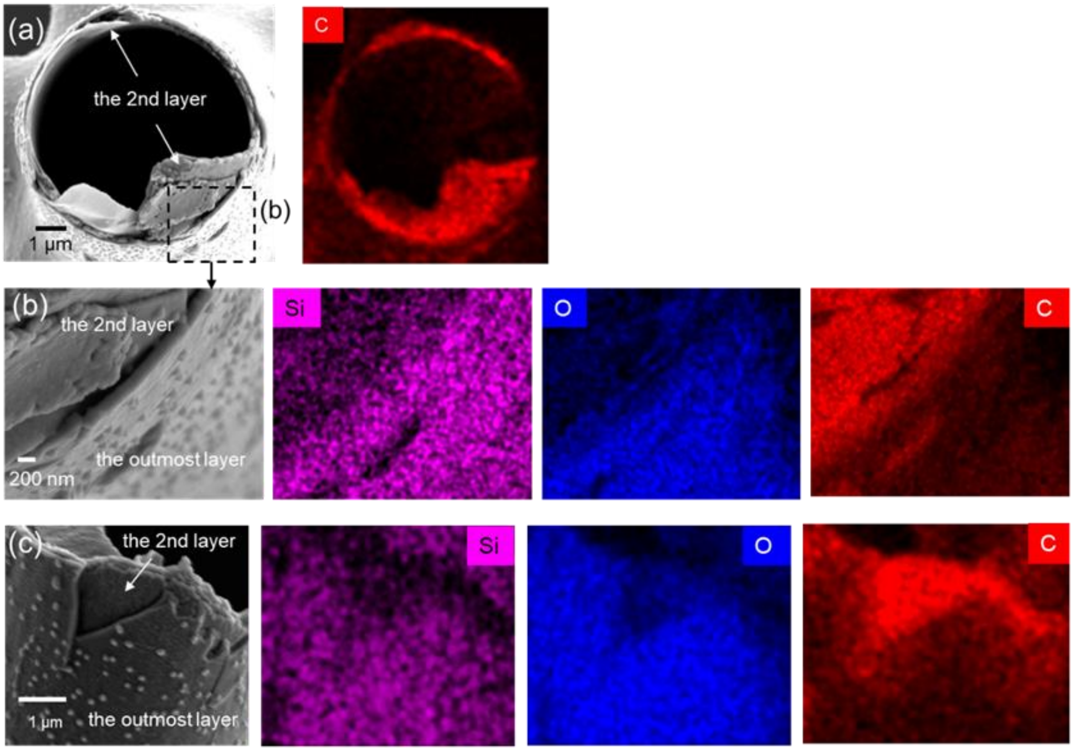

Figure S11. SEM and EDS images of a cross section (a, b) and a side view (c) of a radial spine. The outmost layer consists of silica. The second layer contains a large amount of carbon.

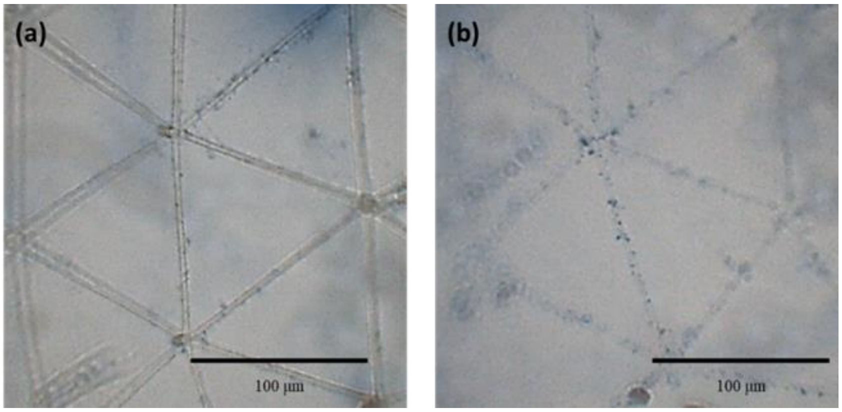

Figure S12. Optical microscope images of the framework after Coomassie brilliant blue staining (a) and (b) after subsequent HF treatment.
